# Biomechanical analysis of the human derived soft tissue graft Epiflex for use in oral soft tissue augmentation

**DOI:** 10.1186/s40729-024-00534-1

**Published:** 2024-03-22

**Authors:** Keyvan Sagheb, Robert Noelken, Saskia-Vanessa Schröger, Christian Walter, Julian Jakob Graef, Sven Schumann

**Affiliations:** 1 Department of Oral and Maxillofacial Surgery, University Medical Center of the Johannes Gutenberg-University Mainz, 55131 Mainz, Germany; 2 Institute of Anatomy, University Medical Center of the Johannes Gutenberg-University Mainz, 55131 Mainz, Germany; 3 Private Practice for Oral Surgery, Lindau/Lake Constance, Germany; 4 Oral- and Maxillofacial Surgery-Facial Plastic Surgery, Mediplus Clinic, Haifa-Allee 20, 55128 Mainz, Germany

**Keywords:** Epiflex, Acellular dermal matrix, Implantology, Soft tissue augmentation

## Abstract

**Purpose:**

This study aimed to investigate the biomechanical properties, cell migration, and revascularization of the acellular dermal matrix Epiflex. As a decellularized, freeze–dried human skin graft, Epiflex has broad applications in medical fields, particularly in implantology and dentistry. Understanding its biomechanical characteristics is crucial for its clinical adoption as a novel soft tissue graft option.

**Methods:**

Epiflex (*n* = 3) was evaluated in comparison to palatal tissue from body donors (*n* = 3). Key metrics, such as elongation and tear resistance, were quantified. Both graft types underwent histological analysis and scanning electron microscopy. Additionally, the healing properties of Epiflex were assessed using a Chorioallantoic Membrane (CAM) Assay.

**Results:**

Biomechanically, Epiflex (mean = 116.01 N) demonstrated the ability to withstand greater forces (*p* = 0.013) than human palatal tissue (mean = 12.58 N). When comparing the elongation, no significant difference was measured (ASG mean = 9.93 mm, EF mean = 9.7 mm). Histologically, Epiflex exhibited a loosely connected network of collagen fibers with a dense upper layer. The CAM Assay indicated efficient revascularization.

**Conclusion:**

Epiflex appears to be a viable option for soft tissue augmentation, particularly appealing to patient groups who avoid all or specific animal-derived products due to ethical or religious reasons.

**Graphical Abstract:**

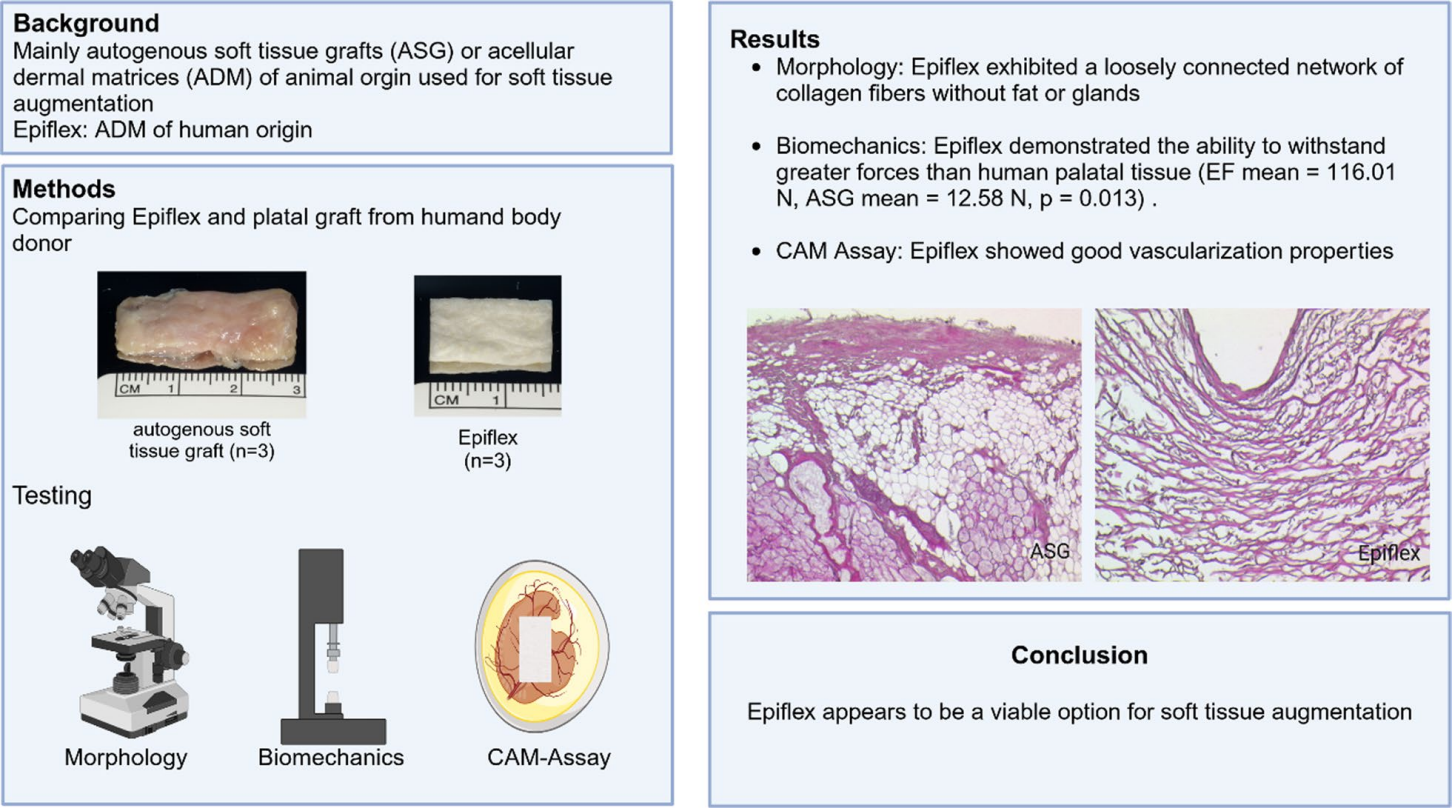

## Background

In dentistry, regeneration of soft tissue is often a necessary step when performing implantology or surgical assisted periodontics. Currently autogenous soft tissue grafts (ASG) or acellular dermal matrices (ADM) are used. The advantages and disadvantages of both grafts are up for debate [1].

ASG are recognized for their healing properties and reliable results, therefore seen as gold standard in soft tissue augmentation. Nevertheless, ASG come with some noteworthy associated risks. Palatine soft tissue is a limited resource, therefore requiring a careful decision where ASG is the most useful. Harvesting ASG prolongs the surgery and patients regularly show complications like pain, swelling, bleeding and infections at the donor site [2].

ADM is decellularized skin mainly harvested from animals, but products from human origin are also available. This procedure preserves the extracellular matrix and enables the migration and revascularisation of the membrane. ADM were developed for the treatment of severe burnings [3], and later adopted for breast reconstruction, plastic surgery and dura mater replacement [4].

ADM are regularly used in dentistry for root coverage and soft tissue augmentation. ADM come with their own disadvantages. Histomorphometrical studies show higher shrinkage rates compared to ASG through the lack of reepithelization [5].

In Europe, many commercially available ADM products are of animal origin, with limited options for human-derived products. Epiflex, a decellularized freeze–dried human skin graft, represents one such ADM. With an increasing number of individuals adopting vegan or vegetarian lifestyles [6] due to concerns about animal welfare [7] and religious believes restricting the consumption of animal origin, these patients require alternatives that are equally reliable, safe, and predictable. Especially porcine derived xenografts are refused by patients mainly for religious reasons [6] in Muslim predominant communities.

This study aims to investigate the biomechanics, cell migration and revascularisation properties of Epiflex and compare it to the palatine connective tissue graft. This should help to determine if Epiflex is a viable option in soft tissue regeneration.

## Materials and methods

### Samples

**Fig. 1:**
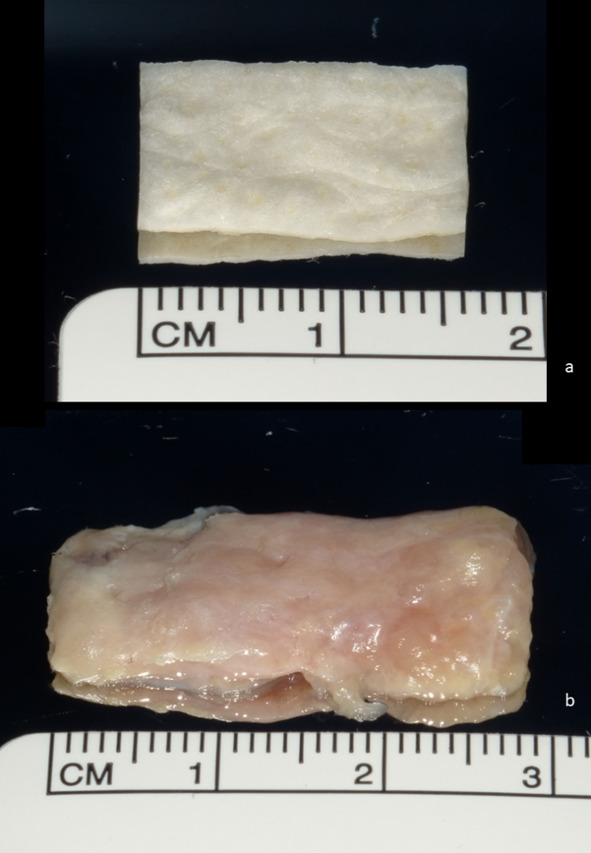
**a** Epiflex before testing; **b** autologus soft tissue graft after harvesting, before testing

In this project we compared autogenous soft tissue grafts (ASG) to Epiflex (EF; Deutsches Institut für Zell- und Gewebeersatz gGmbH, Berlin, Germany). EF is harvested postmortem from screened donors and later decellularized, sterilized and preserved. For testing it is rehydrated with 0.9% saline solution (B. Braun Melsungen AG Melsungen, Germany) according to the manufactures protocol (Fig. 1).

ASG was gathered from two body donors’ hard palate as connective tissue graft (30 × 15 mm, *n* = 4) using standard surgical protocols considering the anatomy of the palatine artery and nerve and the distance to the upper molars.

In accordance with common donation procedures for Anatomical Institutes in Germany, the bodies were donated for medical education and research. After the death and transfer to the Institute for Anatomy, the bodies were stored frozen at − 20° C and later defrosted for collection of the ASG. The thickness of the ASG was controlled by a sliding calliper. The ASG was used without any further processing. Both ASG and EF were cut in a rectangular shape (15 mm × 10 mm).

### Biomechanical testing

ASG (*n* = 3) and EF (*n* = 3) biomechanical properties were evaluated with a material test machine (model 5942; Instron, Pfungstadt, Germany) and the software BlueHill (version 2.25; Instron). Each graft was put between the opposing brackets and draged uniaxial with a constant strain rate of 0.5 mm/s under displacement control until rupture. The maximum load (ML, Newton [N]) and the expansion (E, [mm]) were measured [7].

### CAM-assay

To analyse angiogenetic properties the CAM-Assay was used to histologically evaluate vessel growth and proliferation. Epiflex was cut in a rectangular shape (10 mm × 20 mm) and placed on the chicken embryo for up to 24, 72 and 120 h. Afterwards the membrane was harvested and prepared for histology. ASG was not used due to its body donor origin and in literature well demonstrated healing properties. Since grafts from body donors were obtained post mortem and start to decompose shortly after thawing, it was impossible to perform CAM-Assays with them.

### Histology

Native ASG, EF and CAM-Assay samples were fixed in buffered formaldehyde solution (4%) and embedded in paraffin. Staining of native ASG and EF was performed with hematoxylin and eosin (H&E), Heidenhain’s Azan, Sirius red and Weigert’s elastic according to standard protocols. Briefly, sections were deparaffinized in xylol and rehydrated in ethanol of decreasing concentrations. H&E staining was performed with hematoxylin (Sigma-Aldrich, St. Louis, Missouri, US) and eosin (Sigma-Aldrich) after flushing in distilled water. For Heidenhain’s Azan staining, a staining kit was used (Cat No. 12079, MORPHISTO Ltd., Offenbach am Main, Germany). Sirius red staining was conducted using Sirius red F3B (Chroma, Waldeck GmbH & Co. KG, Münster, Germany), and picric acid (Sigma-Aldrich). For Weigert’s elastic staining resorcin-fuchsin (Chroma) and nuclear fast red (Sigma-Aldrich) were used. After staining, a dehydration of ethanol in increasing concentration and treatment in xylol followed. CAM-Assay samples were H&E stained as mentioned above. Vascular endothelium was stained with an immunoperoxidase reaction. Briefly, 7 μm thick sections were deparaffinized in xylol and rehydrated in ethanol of decreasing concentrations. After flushing in distilled water, slices were protein blocked and incubated with a polyclonal mouse antibody against CD31 in an 0.015 mol/L sodium azide overnight at 4 °C, followed by incubation with biotinylated anti-mouse IgG (1:200 in PBS/5% BSA) for 30 min at room temperature. The Vectastain® Elite ABC kit for peroxidase (Vector laboratories, Burlingame, California) was used, according to the protocol of the manufacturer, for signal enhancement. Detection was carried out with 3,3′diaminobenzidine (DAB, Sigma, St. Louis, Missouri) as chromogen. Nuclear staining was performed with hematoxylin (Roth, Karlsruhe, Germany). After staining, slides were dehydrated in ethanol of raising concentrations and embedded in Eukitt® (Sigma). Negative controls were performed by omitting the primary antibody on consecutive sections.

Histological sections were imaged with a Leica MS 5 tripod (Leica Microsystems, Germany) and a JVC KY-F75U C mount digital camera (JVC, Yokohama, Japan).

### Scanning electron microscopy

After tensile strength measurement ASG and EF were prepared for scanning electron microscopy (SEM). The samples of both grafts were fixed in 2.5% buffered glutaraldehyde solution, washed in phosphate buffered saline (PBS), dehydrated in ethanol, freeze–dried, mounted on specimen holders, sputtered with gold in an argon atmosphere, and visualized with a scanning electron microscope (SEM; ESEM XL-30, Philips, Eindhoven, Netherlands).

### Statistics

Statistical analysis was performed with IBM SPSS Statistics 27 (Armonk, USA) using the Mann–Whitney-U-test. P-values below or equal to 0.05 (*p* ≤ 0.05) were deemed statistically significant.

## Results

### Histology

**Fig. 2:**
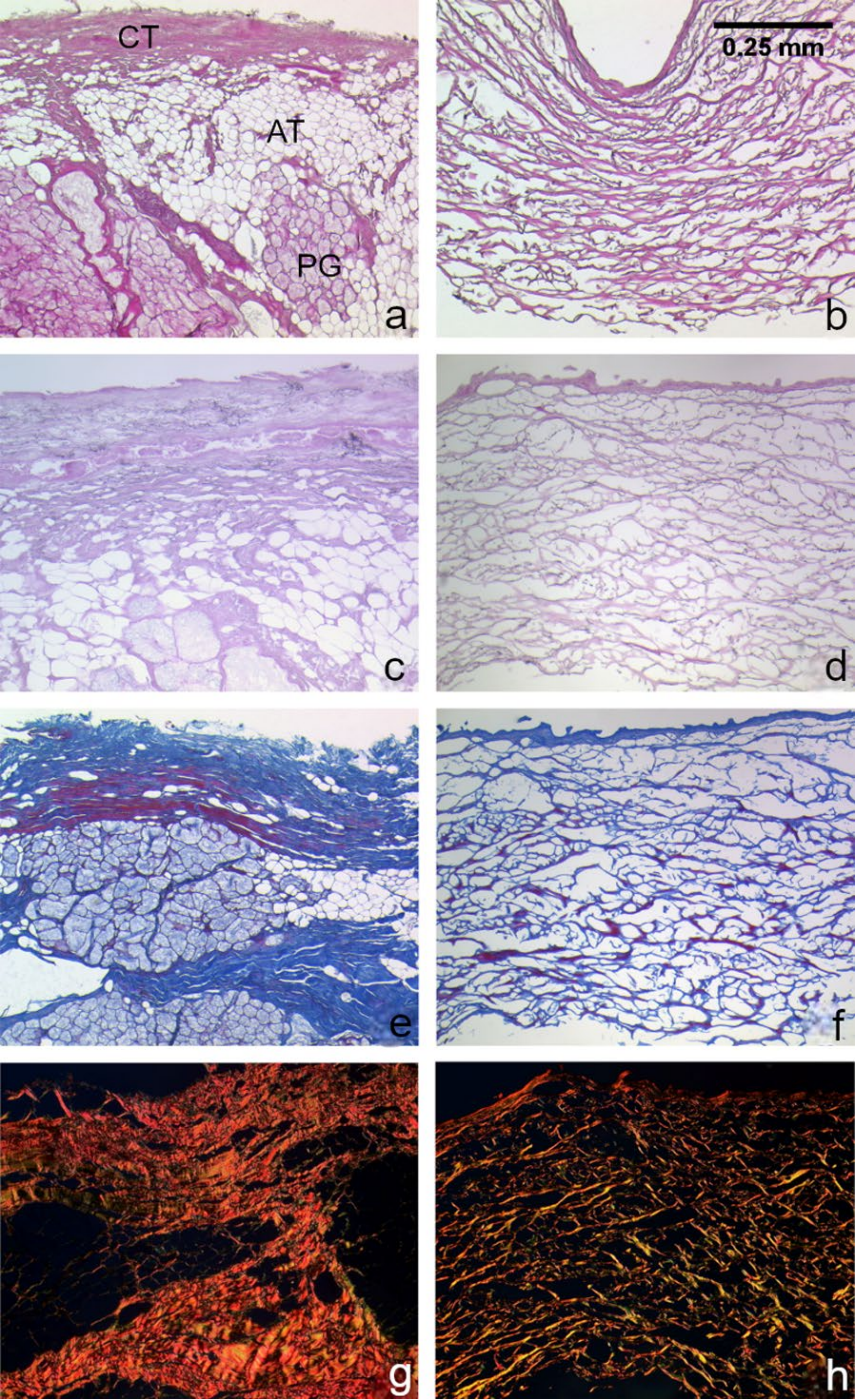
H&E stained ASG (**a**), H&E stained EF (**b**), Weigert stained ASG (**c**), Weigert stained EF (**d**), Azan stained ASG (**e**), Azan stained EF (**f**), sirius stained ASG (**g**), sirius stained EF (**h**)

H&E stained ASG (Fig. 2a) shows the characteristics of palatine oral mucosa, like the dense connective tissue (CT), adipose tissue (AT) and palatine salivary glands (PG). The H&E stained EF (Fig. 2b) shows an outer densely packed collagen layer. Internally the connective tissue is more loosely packed but aligned with fibres parallel to the outer layer.

Comparing both Elastica stained tissue graft samples, the ASG (Fig. 2c) shows a more tightly packed CT layer with unaligned elastic fibres. The dense layer of EF (Fig. 2d) contains no elastic fibres. EF inner layer mainly contains collagen fibres.

Azan-stained samples of ASG (Fig. 2e) show blue coloured collagen with parts of red stained muscle in CT. EF (Fig. 2f) comprises blue stained collagen with read stained sections associated with the fibres, indicating the presence of muscle fibres.

The Sirius red stained samples of ASG (Fig. 2g) are largely coloured red-orange collagen fibres, which indicates the presence of collagen type I. In fewer amounts green fibres are visible which indicates the presence of reticulin fibres. EF (Fig. 2h) shows a dens red layer of collagen and a loosely inner section of red-yellow stained collagen both of type I. Here green dyed reticulin fibres are also visible.

### Maximum load and elongation

**Fig. 3:**
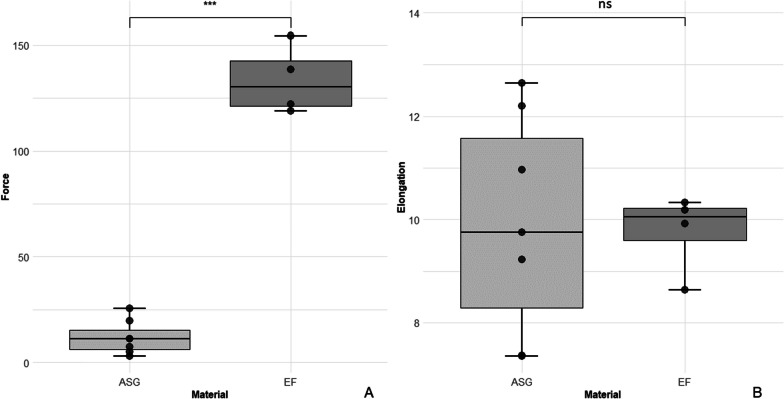
**A** Maximum load of tear forces withstood by ASG and EF. A significant difference (***p = 0.013) between ASG (mean = 12.58 N) and EF (mean = 116.01 N) was observed. **B** Elongation of ASG and EF under tear forces. No significant difference between both groups could be witnessed

ASG showed a significant decreased maximal load compared to EF (*p* = 0.013). The mean value of EF (mean = 116.01 N) compared to ASG (mean = 12.58 N) was more than nine times higher (Fig. 3).

The mean expansion of ASG (mean = 9.93 mm) and EF (mean = 9.7 mm) showed no significant difference in their length increase while expanding (Fig. 3).

### Scanning electron microscopy

**Fig. 4:**
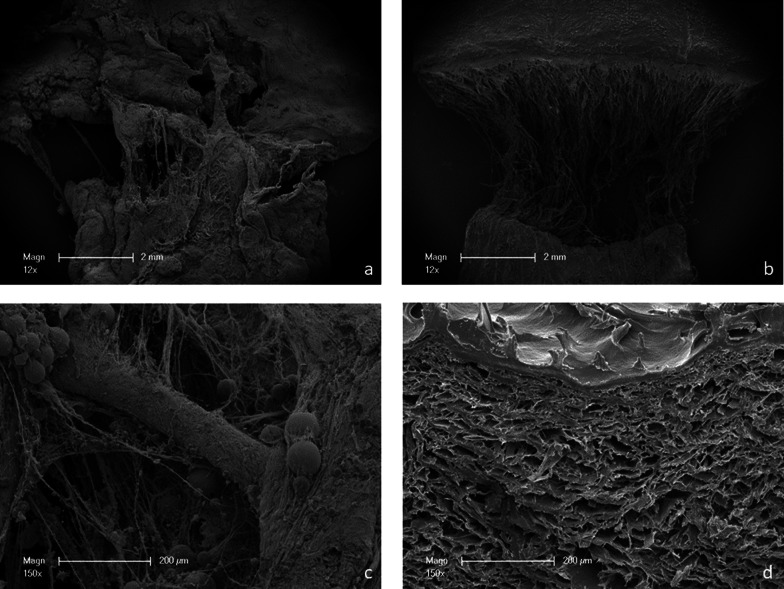
ASG (**a**, **c**) and EF (**b**, **d**) images captured with a Scanning Electron Microscope at ×12 magnification (**a**, **b**) and ×150 magnification (**c**, **d**). EF (**b**) shows a clear cut-off edge with multiple fibers of the connective tissue layer still being connected. With a grater magnification EF (**d**) shows dense 20 μm thick upper layer. Underneath a network of fibers is visible. ASG (**a**) shows an inhomogeneous tear of edge. Some fibers are partly connected. ASG (**c**) incorporates glands which are incorporated in the connective tissue layer

The different properties described in “Histology” section can also be seen in the SEM. EF (Fig. 4b, d) has a dense upper layer of around 20 μm thickness. The underlying collagen is structured as a spongious network surrounding empty areas which formerly incorporated cells and other elements of the extra cellular matrix.

Looking at EF tear-off edge (Fig. 4b), the dense upper layer shows a nearly clean cut with just minor fibers stretching in the gap. The lower layer is still partly connected. ASG (Fig. 4c, d) is more densely packed. Below the CT layer parts of the palatine salivary glands can be seen surrounded by collagen fibers (Fig. 4c). The tear-off edge (Fig. 4a) is not as clean and straight compared to EF.

### CAM-assay

**Fig. 5:**
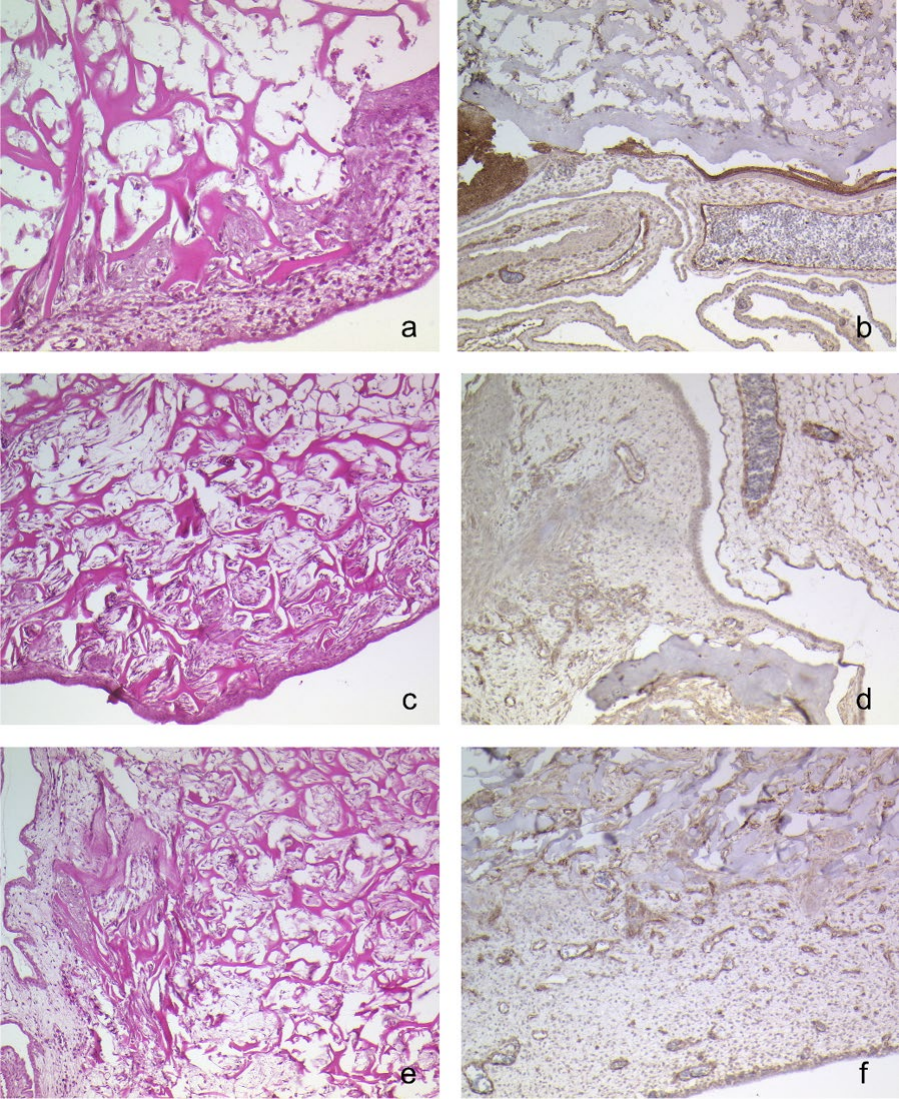
H&E stained (**a**, **c**, **e**) and CD31 immunohistochemistry stained (**b**, **d**, **f**) sections of EF on the CAM. An increasing proliferation of cells and the revascularisation after 24 (**a**, **b**), 72 (**c**, **d**) and 172 (**e**, **f**) hours can be witnessed

After 24 h cells migrated into the membrane but only occupied the upper layer beneath the epithelium. In the CD31 immunohistochemistry image (Fig. 5b) only a few forming vessels can be found.

The 72 h (Fig. 5c) section shows even more cells migrating. The CD31 stained images after 72 h show an increase of vessels.

On the 5th day cells penetrated EF fully. The upper dense layer is still intact. Underneath many cells reorganized the ECM, changing the original structure of the connective tissue layer. The vessel structure grows (Fig. 5f) building sinusoids.

## Discussion

In this study we demonstrated a significant difference between the biomechanical properties of ASG and EF. Despite both being of human origin, EF is significantly more resistant against tear forces.

The difference between ASG and EF could be attributed to the type of tensile, their alignment, and their layer thickness [8].

EF contains as a split thickness skin graft an epidermal layer and a portion of the dermis [9, 10]. ASG lack an epidermal layer but only contain a thin layer of connective tissue followed by salivary glands.

Mainly Collagen is present in ASG and EF and is responsible for much of the materials resistance to tear forces [11]. It is the most abundant collagen and present in wound healing, tendons and the dermis [12].

In ASG and EF, collagen type 1 is located densest in the upper layer of both materials.

Therefore, the densely packed epidermal layer of EF probably provides the most resistance to the tear forces. This assumption is supported by looking at the SEM images. The loosely connected connective tissue layer in contrast to the near clean cut of the epidermal layer indicates that most of the force was absorbed by the epidermis. The dense packing of collagen fibers and their alignment is the main contributing factor for resistance to tear forces [13].

In ASG the connective tissue layer contains the densest Collagen packing. When comparing the SEM images ASG also shows the shear point in its densest collagen layer. Underneath, the salivary glands and their surrounding supporting fibres are still partly connected, as in the connective tissue layer of EF. This fuels the assumption that the force resistance of both materials is retrieved from the dense collagen layers, the connective tissue layer in ASG and the dermal layer in EF.

When comparing the upper layers of both materials a different morphology can be witnessed. EF shows a thin but densely packed and clearly parallel aligned tensile structure. ASG in comparison is packed lighter and the fibres are not as well aligned. This could be attributed to the origin of ASG and might explain the greater variance in the elongation tests compared to EF. The function of the connective tissue of the palat is the adherence to palatine bone.

This contrasts with the epidermis, where part of its function is the resistance to external mechanical forces [14].

Biomechanically EF seems to be the better solution than ASG. Comparing EF with commonly used porcine or bovine tissue grafts comparable values (NovoMatrix, mean = 144.68 N) or lower values (mucoderm, mean = 57.91 N) can be witnessed [15]. These materials are established products for oral soft tissue regeneration and deliver reliable results, but their advantages over autologous soft tissue crafts are up for debate [16, 17]. The main advantages of acellular dermal matrices are their ease in handling and reduction of surgery time. Post-surgery complications like pain, bleeding and infection would be eliminated and therefore increase patient satisfaction [18]. Studies show that there is no significant difference between human and animal ADM in biocompatibility [19].

Information about the healing properties and complication rates for EF are limited for oral use. Animal trials of allogenic freeze–dried skin on gingivae of monkeys showed the trouble-free incorporation of the material and a reduced immunologic response [20]. The CAM-Assay shows that the incorporation of EF is not hindered by its histological structure. Cells can migrate easily, and revascularisation enables the full integration in the surrounding mucosa.

EF is commonly used in breast reconstruction and shows complication rates comparable to other acellular dermal matrices [21]. In trauma surgery EF was used for bone-defect regeneration and showed comparable results in rats to the currently used technique [22].

All these results indicate good healing properties and make EF a reasonable choice for oral soft tissue regeneration. Further studies should aim to better understand the healing properties and interaction with oral mucosa, especially when used for bone augmentation and implantation.

A limiting factor for the widespread use of EF in Implantology could be its human origin and therefore limited supply. EF is labelled for many different cases such as skin replacement after severe burnings, breast cancer therapy, hernia therapy and many more [23]. The supply is limited to body donors who meet the selection criteria such as age below 75 years, cause of death, medical history and storage conditions of the donor body. Then the donors are screened for infectious diseases like HIV, Hepatitis A, B, C, and Syphilis [9]. This limits the production of EF to the number of donors who meet the selection criteria. The availability is restricted even more through its widespread use in other medical fields.

On the other hand, EF could be a reasonable choice for patients who dislike the use of specific animal products for religious or ethical reasons [24]. Religious beliefs which limit the use of certain animal products are widespread in European society [6, 25]. Ethical considerations are also a growing factor when using animal derived products [26]. Patients demand more information and open communication by their Surgeon. This includes the use of animal products [24].

Our results indicated that both animal and human derived soft tissue grafts are reasonable choices for soft tissue augmentation. They are superior to autologous soft tissue grafts in decreasing complication rates due to the missing morbidity at the donor site and therefore might contribute to a greater patients’ satisfaction. The lack of a clear advantages of any of the groups (animal, human or autologous) makes the decision for one product dependent of the surgeons’ and patients’ preferences.

## Data Availability

The datasets used and/or analysed during the current study are available from the corresponding author on reasonable request.

## Abbreviations

ASG: Autologous soft tissue graft
ADM: Acellular dermal matrix
EF: Epiflex
CT: Connective tissue
AT: Adipose tissue
PG: Palatine salivary glands
ECM: Extracellular matrix
SEM: Scanning electron microscopy
